# A Framework to Describe, Analyze and Generate Interactive Motor Behaviors

**DOI:** 10.1371/journal.pone.0049945

**Published:** 2012-11-30

**Authors:** Nathanaël Jarrassé, Themistoklis Charalambous, Etienne Burdet

**Affiliations:** 1 Department of Bioengineering, Imperial College of Science, Technology and Medicine, London, United Kingdom; 2 Institute of Intelligent Systems and Robotics, UPMC - University Pierre et Marie Curie, CNRS - UMR 7222, Paris, France; 3 Automatic Control Lab, Electrical Engineering Department and ACCESS Linnaeus Center, Royal Institute of Technology (KTH), Stockholm, Sweden; Bielefeld University, Germany

## Abstract

While motor interaction between a robot and a human, or between humans, has important implications for society as well as promising applications, little research has been devoted to its investigation. In particular, it is important to understand the different ways two agents can interact and generate suitable interactive behaviors. Towards this end, this paper introduces a framework for the description and implementation of interactive behaviors of two agents performing a joint motor task. A taxonomy of interactive behaviors is introduced, which can classify tasks and cost functions that represent the way each agent interacts. The role of an agent interacting during a motor task can be directly explained from the cost function this agent is minimizing and the task constraints. The novel framework is used to interpret and classify previous works on human-robot motor interaction. Its implementation power is demonstrated by simulating representative interactions of two humans. It also enables us to interpret and explain the role distribution and switching between roles when performing joint motor tasks.

## Introduction

**Figure 1:**
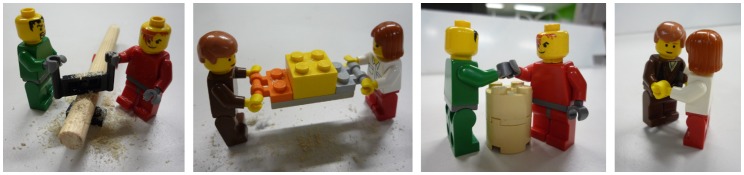
Different tasks requiring interaction between two agents (here represented with Lego® parts and characters). From left to right: sawing, lifting a heavy load together, agonistic arm wrestling task and interactive dancing task.

Joint action is a fundamental aspect of human life [1], as we collaborate or interact with peers in most actions. This paper concerns in particular joint actions with *motor interaction*, which stands either for “physical interaction” (which is ambiguous as physics is not restricted to mechanics) or for “haptic interaction” (as haptics concerns (touch and force) sensing while interaction additionally requires a motor action). Many common tasks rely on the motor interaction of two humans, such as sawing, dancing, physical rehabilitation, fighting, mating, carrying a table, etc. [2] (see some examples in Fig. 1). As voluntary movement is the defining characteristic of animals, it is plausible that motor interactions are at the basis of all social and communication behaviors [3]. How humans deal with motor interactions is largely unknown, and has not been systematically studied until recently. In fact, in the last 150 years, human motor control research has been devoted mostly to the study of walking [4] and free arm movements [5]. It is only in the last 40 years that robotic interfaces have been used to investigate how humans interact with the environment (e.g., [6]–[8]) and with each other (e.g., [9], [10]) to perform a variety of tasks.

Understanding how humans interact in tasks requiring motor interaction is an interesting and challenging new field of research, and is critically important to designing robots interacting with humans. Recent years have seen a surge of cooperative robots, such as assistive devices for industry [11], robotic wheelchairs to increase the mobility of people with physical or cognitive deficits [12], workstations with haptic feedback which can be used to train surgeons [13], and robotic systems to increase the amount and intensity of physical therapy after stroke [14].

Therefore, it becomes necessary to develop tools for characterizing and understanding the nature and the issues of interactive tasks. Having a taxonomy of interaction kinds and strategies would enable us to identify the interaction strategies humans use. This may help us creating robots that react as humans do during motor interaction, as well as efficient human-robot dyads able to use the best of the human and the robot. Therefore, we would like to design *a taxonomy of interactive behaviors that can classify the different kinds of motor interactions, model the agents' behavior and simulate their control*.

In order to do so, we first reviewed literature on motor interaction behaviors in the fields of human computer interaction (HCI), robotics, psychology and game theory [15]. The main results on taxonomies for motor interactions can be summarised as follows:

- Some taxonomies from HCI (e.g., [16], [17]) can be used for motor interactions, but are not specific to them and difficult to apply in concrete tasks.
- Analyses of motor interaction kinds [18], [19] have defined roles according to either the trajectory [20] or the force [21]. Models using both trajectory and force (e.g., [22]) are complex and thus difficult to use.
- A few implementations of controllers with flexible behavior have been developed [20], [21], which are based on simplified taxonomies and thus not adapted to all situations. For instance, important motor interactions for humans such as competition have not been considered.
- While studies on psychological [23] and social factors [24] influencing joint action focused on kinematic and haptic information exchanges present interesting analyses, they could hardly be used to generate joint motor behaviors.

These shortcomings of previous taxonomies for motor behaviors prompted us to describe the role distribution during a joint motor action in a simple quantitative way. First, the nature of the task and how it constrains the choice of possible behaviors for each agent and their interactions was studied. Then, the role of each agent was defined through a cost function that it needs to minimize, and the interaction between the two agents arises by their physical coupling. This enables us to use mathematical tools from Game Theory, optimal control and nonlinear adaptive control in order to derive the two partners' motor behavior and adaptation.

It has been shown in neuroscience studies that humans interact with the environment by minimizing error (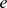) and effort (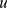) [25], [26], which can be modelled as the minimization of the cost function
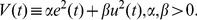
(1)Furthermore, when interacting with novel dynamics, humans adapt force, mechanical impedance and trajectory to minimize such a cost function [27]–[29]. Similar cost functions will be used to model the interaction of two agents.

This paper's outline is as follows. A framework for motor interactive tasks and control is first introduced, in the form of a simple taxonomy for the interaction between two agents, physically coupled (directly or through an external object or tool) and conditioned by the tasks they are carrying out. The paper then presents how the taxonomy can be used to classify existing implementations of human-robot motor interaction, and provides an overview of the problems that remain to be addressed. The new taxonomy can also be used to generate appropriate behaviors, as is illustrated in simulations. Finally, possible applications of the framework to other fields like behavioral psychology and agent theory are described.

## Methods

### A framework for motor interactions

Game theory [30], which describes and analyzes situations where interactive decisions take place, appears as a natural framework to consider the motor interaction in a human-human, human-robot or robot-robot dyad. Game theory comprises a set of analytical tools to predict the outcome of complex interactions among decision makers, obeying to a strategy based on perceived or measured results. Two-player games, such as the motor interactions considered in this paper, play a fundamental role in game theory because their analysis is straightforward; John von Neumann's minimax theorem [30] establishes a unique value of such games.

Models that address the interaction among individual decision makers are called *games* and the rational decision makers are referred to as *agents* in this paper. Interaction between the agents is represented by the influence that each agent has on the resulting outcome through a cost function representing its objectives. Steady-state conditions in which each player is assumed to know the equilibrium strategies of the other players, and no player has anything to gain by changing only his own strategy unilaterally, known as *Nash equilibria*, can be identified [31], [32]. The interaction tasks can be seen as *differential games*, also called *utility-based games*, where the evolution of the partners' state variables is governed by differential equations. The problem of finding an optimal strategy in a differential game is closely related to optimal control. Note that while game theory has been originally conceived to model conscious (and also rational) decisions of agents, interaction behaviors may be at least in part automatic (i.e. without voluntary control) and sometimes unconscious. Agents behavior may be well described by the mathematical (game theoretical) framework without assuming that they know exactly what they have to do or think about it. However, the reaction to a sudden change that can be seen as irrational is considered as a transition in the system so does not affect its properties (such as existence, uniqueness, etc.).

#### Interaction definition

We consider the interaction of two agents, 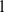 and 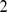, that:

- generally aim at minimizing their effort 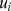, 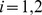.
- perform separate actions 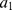 and 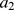 or a common action 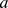, whose performance is rewarded by reinforcement signals 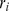 or evaluated through error measures 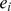, 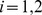.
- are each equipped with multimodal sensors. To simplify the exposition, we will focus on sensors measuring position, force and body contact. Agent 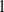 is able to perceive its own error 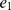 and estimate the partner's error 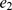 (denoted by 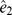), whereas agent 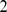 perceives 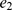 and estimates 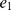 (denoted by 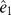).
- are equipped with actuators able to affect the environment and the other agent with suitable force and mechanical impedance, using a controller 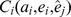, 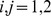, 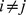 (*i.e.*, 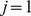 if 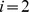 and 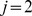 if 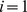). 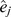 denotes the estimate from 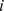 of the error of the other agent.

In summary, each agent 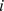 has to fulfil a task by minimizing some error (or maximizing some reward) while using minimal metabolic cost. Each *interaction behavior* will arise from the combination of the minimization of the individual cost functions 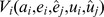, 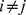, 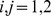, and is thus characterized by these two cost functions.

The nature of the motor interaction between the agents depends on the combination of their personal behaviors, as will be described in more detail in the “*Taxonomy of interactive behaviors*” section of the Methods, and is also constrained by the particular task(s) carried out, which will be described in detail in the “*Divisible vs. interactive tasks*” and “*Agonistic vs. antagonistic tasks*” sub-sections of the Methods. These cost functions can also be used to adapt behavior as will be described in the “*Learning*” paragraph of the Results. The following task description extends the approach of [33] about representations and action monitoring supporting joint action.

#### Divisible vs. interactive tasks

**Figure 2:**
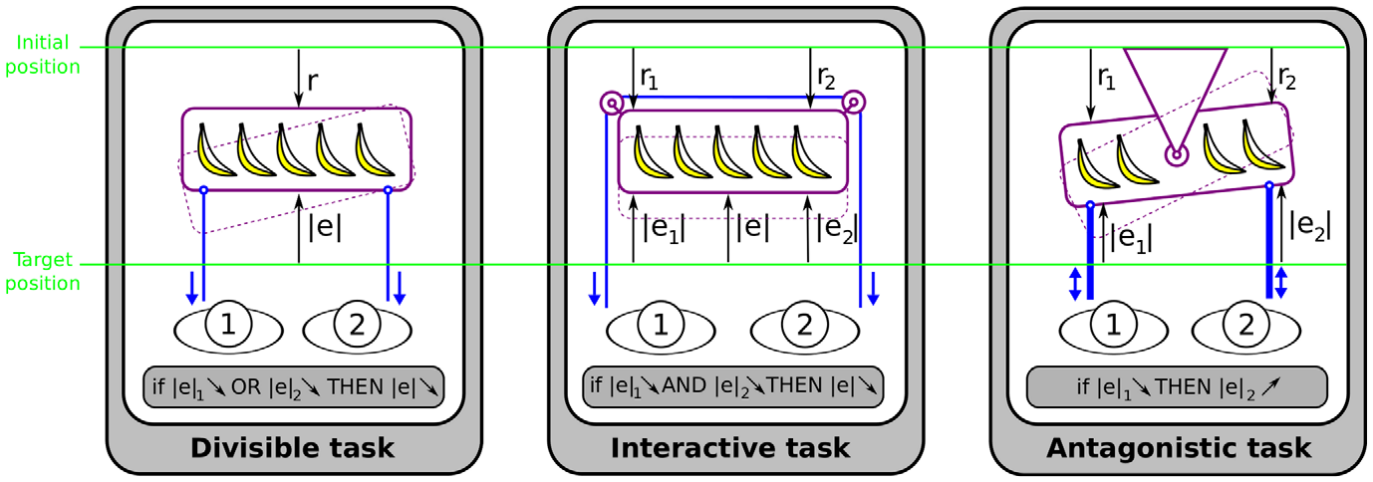
Example of different kinds of tasks two agents can carry out. We consider simple tasks in which two animals can pull a rope in order to approach a pallet with food. 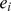 is a measure of error relative to the target and 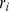 a reward increasing when the state approaches the target. In the *divisible task*, each agent contributes to his own subtask (i.e., pulling the rope), which helps getting the pallet for both animals. In the *interactive task* the two agents have to collaborate in order to succeed in completing the task. In the *antagonistic task* the performance of one agent is detrimental to the other.

We start our description with *divisible tasks*, which are composed of compatible subtasks that can be completed by each agent independently. In some cases the task could be completed by each agent alone, such as painting a house walls together [34], or the task in the left panel of Fig. 2, where two animals can pull a rope to move a pallet and obtain food. Other divisible tasks have disjunct but complementary subtasks, such as a hybrid force-position controller in which position control and force control are executed independently in separate subspaces [35].

In divisible tasks, the two agents do not need to know anything about the other agent in order to succeed in their respective subtask. As the two agents are acting independently, each agent can minimize its own error and effort, which we represent by the same cost function as was found when one human is interacting with the environment [25]:
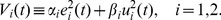
(2)We name such independent behavior *co-activity*.

A task in which (at least) one agent needs a partner to carry out its (sub)task is called *interactive*. The Game Theory formalism embraces interactive tasks, in which the activity of one agent affects the other agent. The middle panel of Fig. 2 illustrates an interactive task that has been used to examine the social behavior of animals such as chimpanzes [36], elephants [37] and hyenas [38]. In this task no animal can succeed in securing the food without the help of its partner. As in an interactive task the agents' behaviors are dependent, thus the behavior is more complex than with a divisible task, and the cost function depends on both agents: 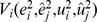, 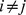. The rich repertoire of behaviors that can be adopted in interactive tasks is described in subsection “*Taxonomy of interactive behaviors*” of the Methods.

#### Agonistic vs. antagonistic tasks

Both divisible and interactive tasks can be agonistic or antagonistic. In an *antagonistic task*, performance improvement in (at least) one agent is detrimental to the partner, due to conflicting interests, as is illustrated in the right panel of Fig. 2. An agent's gain (or loss) of utility is exactly balanced by the loss (or gain) of the utility of the other agent. If the total gains of the agents are added up, and the total losses are subtracted, they will sum to zero; that is why these types of interactive tasks are considered as strictly competitive and correspond to zero-sum games in game theory (the total benefit to both players in the game, for every combination of strategies, always adds to zero). Examples of antagonistic tasks include arm wrestling, rope pulling game and fighting. In general, the agents have distinct subtasks and there is no common task.

In contrary, in *agonistic tasks* improvement in one agent's subtask contributes to the improvement in the common task. This category stains numerous interactive tasks like moving a heavy table together, dancing or mating, where joint action is the only solution to succeed in the task, but also divisible tasks such as hybrid position/force control. Both the left and middle panels of Fig. 2 are agonist tasks. In such a case, the task enforces cooperative behavior and these types of interactive tasks correspond to the cooperative games of game theory.

In summary, tasks are determined by two antagonisms: divisible/interactive, and agonistic/antagonistic. Divisible tasks induce a co-active behavior which will help both agents in agonistic tasks, such as in the left panel of Fig. 2, and can be mutually detrimental in an antagonist task, such as in the right panel of Fig. 2. Similarly, interactive tasks can be either agonistic, such in the middle panel of Fig. 2, or antagonistic, when a Sumo fighter pushes as much as possible against the opponent and suddenly drops the force in order to destabilize him.

### Taxonomy of interactive behaviors

**Figure 3:**
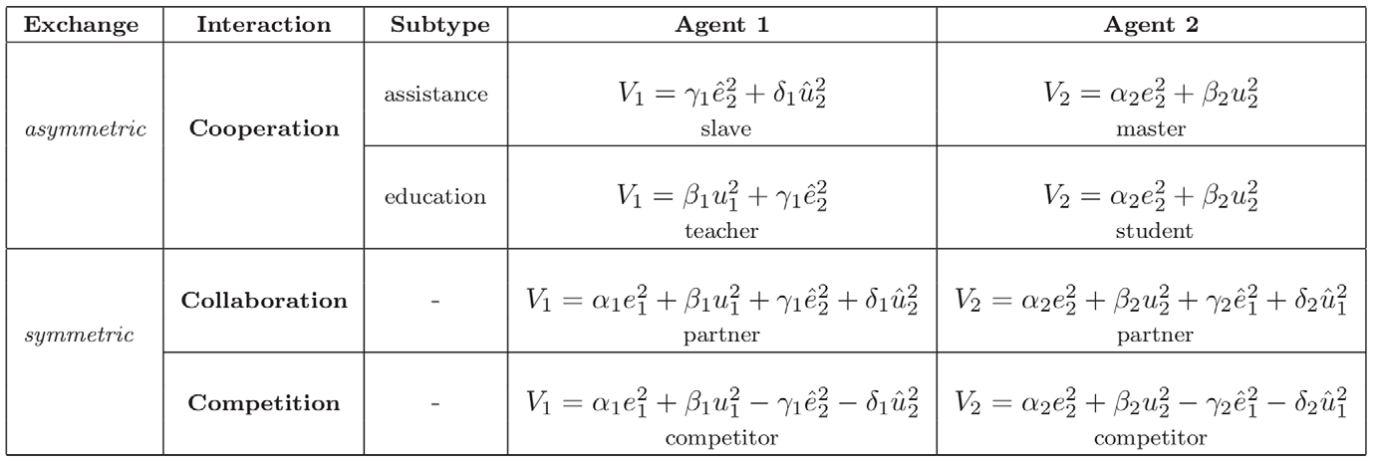
Definition of main kinds of behaviors (in interactive tasks) through cost functions. For simplicity, the time variable 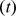 was omitted in the cost functions.

The behaviors adopted to perform interactive tasks can be classified in three main categories: cooperation, collaboration and competition. Competition will be mainly observed during the antagonistic tasks as a noncooperative game, whereas various kinds of cooperation and collaboration will mainly occur during agonistic tasks and will be treated as a cooperative game (the partners are able to form binding commitments). These categories and the associated cost functions are summarized in Fig. 3 and will be described now. Note that the associated cost functions suggest a utility-based game theoretic approach, in which the behavior of the agents depends on the utilities being chosen.

#### Competition vs. collaboration

In a *competition*, both agents focus on their own action and effort, and if necessary impede the other's performance in this purpose:
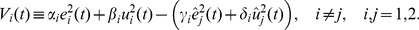
(3)In this scheme the two agents may have different goals, such as reaching different targets at the same time with the same object, or the same goal, such as when two children attempt to grasp the same cookie. In contrast, in a *collaboration* both agents jointly try to develop a consensual solution to solve a problem [39], and, as in cooperative games, no agent has incentive to leave the coalition formed and receive a smaller utility. A collaboration is also modelled as a *symmetric behavior* (i.e., the cost function's structure does not change under the permutation 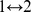), but this time with positive influence on the partner:
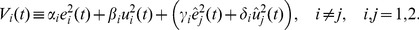
(4)Each agent minimizes its and the partner's error and metabolic cost (i.e., energy, force, etc.).

#### Cooperation vs. collaboration

In a collaboration, there is no a priori roles distribution, but a spontaneous roles distribution depending on the interaction history. Any physical interaction with negotiations and discussions to accommodate others while considering their perspective, belong to this category. In this case “activity is synchronized and coordinated in order to build and maintain a shared conception of a problem” [40].

In contrast, a *cooperation* occurs when different roles are ascribed to the agents prior to the beginning of a task and this distribution is not questioned until its completion. While in collaboration the agents work on an even basis, cooperation has an uneven distribution of subtasks or roles during the task [39]. Cooperating agents work towards the same end and need each other to complete the task, but are not equal. In fact, cooperation is characterized by an *asymmetric behavior*, in the sense of asymmetry in the cost functions as tested from the permutation 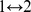.

#### Master-slave vs. education

The most typical asymmetric relationship of a cooperation is the *master-slave* scheme. This behavior is characterized by the following cost functions:
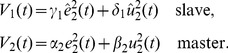
(5)The master is only considering himself, while the slave considers only (his perception of) the master needs. The above cost functions illustrate the danger of this relation, where the slave does not consider its own effort expense and may eventually lose all its energy.

We want now to examine the teacher-student relationship. This relation is critical to human society and education, and also to developing service robots. The efficiency of all kinds of virtual reality based training systems (for surgery, sport, etc.) and robot-assisted physical rehabilitation systems will namely depend on a suitable interaction behavior. One may a-priori think that the master-slave scheme applies here as well, with the teacher as master and the student as slave. However, efficient learning schemes suppose that the student is building his own capacities while the teacher is assisting this process. Similarly, 20 years of experience with robot-assisted neurorehabilitation of stroke patients have shown that stroke survivors improve their motor functions only when actively attempting to move, but do not improve when they can rely on the robot to move their arm [41], [42].

Therefore, the master-slave interaction behavior is not appropriate for education. However, an altered version of an assistance can be considered for the relationship between a teacher and his student, or a sportsman and his coach. A good teacher will try to maximize the student's independence. Therefore, the teacher can minimize his own effort in order to challenge the student, let him perform according to his capabilities and eventually increase them. In the *education behavior*, the cost functions 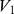 of the teacher and 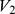 of the student are thus defined as:
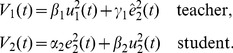
(6)


This definition describes the main quality of a good teacher as the capability to maximize student involvement and action. Even if the teacher is an expert in the task (good at minimizing goal error) or wants to help the student, he should not care too much about the task achievement (i.e., adopt the slave role), but let the student try and improve his or her performance. Indeed, “the goal of the teacher is to become obsolete as soon as possible, leaving the pupil to perform the skill on his or her own” [43].

#### Mutual assistance

Finally, the anecdotical mutual assistance or *reciprocal altruism* [44] can also be represented in our taxonomy, using cost function
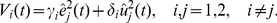
(7)This ideal interaction behavior occurs in particular contexts such as the iterative prisoner dilemma and associated strategies such as tit-for-tat [45], where the interaction strategy is selected by considering long term benefits.

#### A tool to interpret switchings between interactive behaviors

**Figure 4:**
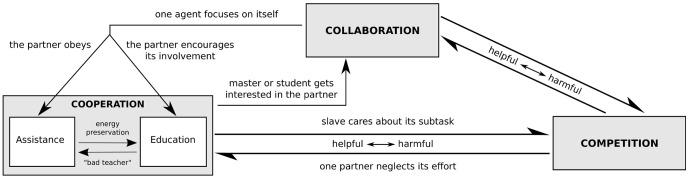
Relations between distinct interaction behaviors. Such changes are mainly controlled by a higher control layer influencing the choice of the interactive strategy, before the interaction (according to some previous experience of the task completion and learning processes) or during the interaction (through the perception of signals that the central nervous system tries to recognize and interpret in order to predict future action.)

The importance of transitions between distinct behaviors has been emphasized in [46]. The above framework enables us to understand the relations between distinct interaction behaviors in the case of interactive tasks. As illustrated in Fig. 4, collaboration and competition both involve symmetric behaviors between the partners and distinguish themselves principally by the helpful vs. harmful interaction, i.e., only by a sign change in the cost function. This may suggest how easy it is to switch between these two interactive behaviors, i.e., from ‘love to hate’ or conversely.

As already mentioned, assistance and education differ only in the energy preservation making the slave an educator, as in Beaumarchais' “Marriage of Figaro” [47], when a clever servant is in fact leading the action and helping the master to change his perspective, which eventually results in a new collaboration. Conversely, a collaboration degenerates into a cooperation when one agent focuses on itself and the other, either obeys in the assistance or accepts to look for the other's task in the education.

Note that both collaboration and competition require from the agents the capacity to interpret their partner's behavior [48]. Therefore, an autistic agent, which may not possess this capacity, would hardly be able to work in a symmetric collaboration or competition situation. In fact, the interaction of two autistic agents may correspond to co-activity. In a cooperation, an autistic agent could be the master or the student (thus is able to learn), while the complementary roles of slave and teacher would require the capability to interpret the partner's behavior.

## Results

### Classification of human-robot interactions

**Figure 5:**
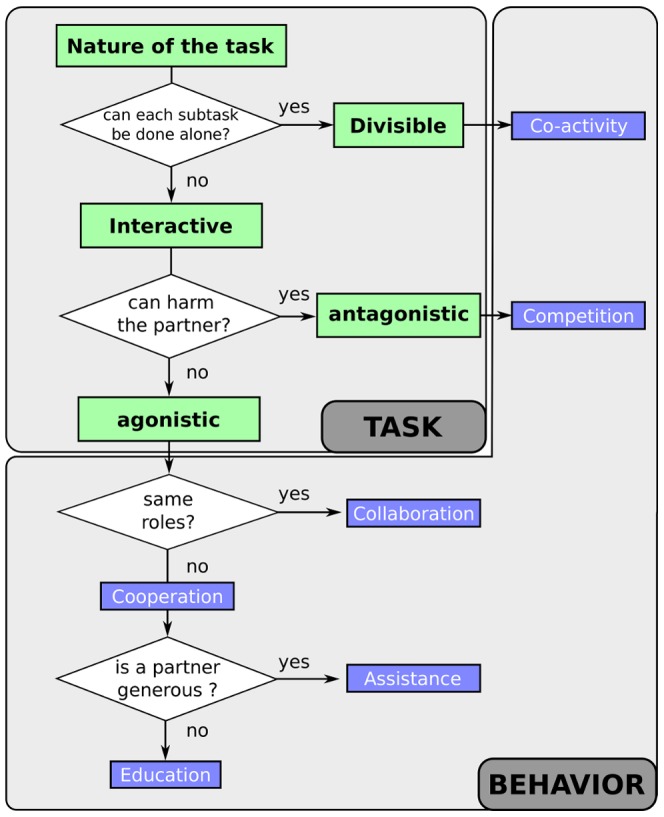
Determination of motor interaction behavior. First the underlying task is determined (green squares). For interactive tasks then the exchanges between the agents determine the type of interaction behavior (blue boxes).

We now want to examine how human-robot interactions can be classified and interpreted within our framework. Based on the analysis of last two sections, we first developed a logigram to facilitate the classification, which is shown in Fig. 5. Note that some questions could be asked in a different order, e.g., first those about the agonist/antagonist, and then those about the divisible/interactive alternatives. This scheme is used to classify various human-robot interaction behaviors found in the literature.

#### Assistance (cooperation)

The name “robot” stems from slave or serf [49], and in fact many projects have developed robotic slaves for assisting humans in performing tasks, e.g., to help lifting and carrying heavy or bulky objects. The most common example is provided by teleoperation systems [50] as well as force extenders or exoskeletons to amplify the physical capabilities of humans [51]. A force amplifying exoskeleton tries to minimize the human master effort, while it is mechanically connected to the human body and is transferring power to it (in contrast to remote teleoperation). Recent years have seen the development of lower limb extenders, in particular for military applications [52].

Robots can be designed to assist human beings in specific tasks by providing assistive forces or trajectory corrections [53], or by guiding movements within a restricted workspace [12], [54]. A robotic interface to guide the user's motion along desired directions while preventing motion in undesired directions or regions of the workspace [55] can be considered as a slave, because it provides appropriate support during action and cannot complete the main task alone. In robotics literature, such robotic aids are encountered as *intelligent assistive devices* (IAD) or simply *robotic lifting assistants*.

Robots providing an assistance behavior also include the *cobots* or *collaborative robots* described in [56]. Despite their name, these robots do not collaborate in the sense of our taxonomy, but are in fact conceived to yield a master-slave behavior. As these cobots track human operator behavior and react accordingly (with for example, a load lifting assistant fitted with an assistance to motions in the plane, provided according to the angular movements of the loading cable [57]), they implement assistance behavior rather than co-activity.

Various platforms, e.g., mobile robots with a robotic arm [58], which involve a controller to detect the intentions of the human user [59], [60] or the control of multiple slave robots [61], are other applications of the assistive scheme. Finally, robot teach pendants where the human teacher directly moves the robot that records the motion to reproduce, or imitation learning [62] where the robot is moved according to data of human movement recorded in some other way, also correspond to an assistance scheme, because the robot is passively following the human example.

#### Education (cooperation)

As mentioned in subsection “*Taxonomy of interactive behaviors*” of the Methods, a typical example of the education type of interaction is the therapist-patient relationship in physical rehabilitation. For instance, during poststroke neurorehabilitation [14], a therapist will help the stroke survivor to move the arm or the hand adequately, but will decrease motion assistance while the recovery progresses. Haptic interfaces for sport training and rehabilitation robots aim at emulating this behavior. Even if the “passive mode” used in first stage, where the arm is moved by the robot, is similar to an assistance scheme, the “active mode” in which robot is only correcting patient movements “just-as-needed” is similar to an education scheme [41], [42], [63], [64].

In fact, a recent model of motor learning in humans provides a suitable tool to adapt assistance provided in rehabilitation robots and sport trainers. In this model [25], [65], [66], force, impedance and trajectory are adapted to minimize motion instability, error and effort. Error minimization ensures that the task will be performed successfully if the human user is not able to do so, but effort minimization makes the robot ‘lazy’ so that the human has to do as much as he or she can. Interestingly, the computational model, based on the gradient descent of a cost function similar to Equ.(1), yields an efficient adaptive controller [28] (briefly described in the *Learning* section of the Results) that can be implemented on rehabilitation and sport robots [67]. Assuming that the patient focusses on his or her performance, he or she will, together with the robot trainer, perform according to the education behavior of Equ.(6).

Educational interaction, where robot is active and corrected by the teacher through motor interaction can further be found in [68] where the user helps a humanoid robot reproducing a movement (previously recorded) to refine its gesture by kinaesthetic teaching, or in [69] where a robot learns how to perform a collaborative manipulation task through demonstration using a haptic interface. Similarly, Ikemoto *et al.* [70] developed an algorithm dedicated to robot learning through physical interaction with humans.

#### Co-activity

There are many divisible tasks where robots or humans interact without needing to know what each other is doing, and incidentally interact and succeed in the common task. In fact, separating tasks in independent but complementary subtasks where each of the robot or the human performs well, is in many cases an efficient way to perform joint actions, as no negotiation thus sensory exchange is required, enabling safe and simple solutions without inference.

For example, the Acrobot robot assistant for bone surgery [71], which constrains surgeon's motion to a predefined region, facilitates surgery without knowledge of the surgical task. Such situations typically arise when the task is decomposed into subtasks carried out by independent controllers. Similarly, simple assistive devices developed to help manufacturing, e.g., to compensate gravity during tool or parts manipulation, use co-activity, as they just compensate load in the vertical direction using actuators or spring systems while leaving the movements on the plane unrestrained.

Some robots that at first sight appear to rely on a competitive scheme, are actually only using a co-activity scheme, and lead to a fight between the partners because of the divisible and antagonistic nature's task. For example, the electroactive polymers (EAP) actuated arm robot [72] that was able to win a wrestling match against a human opponent for the AMERAH challenge (Arm wrestling Match of EAP Robotic Arm Against Human) only tries to minimize its own error without considering human action.

#### Collaboration

A very limited number of projects have tried to implement interaction beyond simple cooperation, by introducing role switching and continuously adapting interaction, thus allowing robots to collaborate with humans. Collaboration examples include the experiments reported in [20], during which robot behavior is continuously adapted to the human partner, and the study [21], where role distribution is negotiated.

Recent work has presented a method in which the robot's assistance level, and thus also its role, are continuously adapted according to an estimate of human's disagreement level [73] or to the magnitude of the partner's contribution, together with a formal analysis of human-robot force cooperation [74]. Another collaboration example consists of implementations of hand-shaking with a robot, because handshake is typically mutual (as illustrated by the fact that a weak and passive hand is felt as weird). A hand shaking robot system providing realistic experiences was developed using a hidden Markov model-based approach that allows the robot to estimate human intentions and adapt its behavior [75].

#### Competition

It is hard to find examples of motor competition between humans and robots in the literature. We believe that this is due to the taboo (as expressed by the first Asimov's laws of robotics) that “a robot may not injure a human being or, through inaction, allow a human being to come to harm” [76]. This has limited research on the development of controllers designed to physically beat humans, while robots are already superior to humans in chess playing [77] and obviously in memory.

While some studies have been made about robot-robot competition, such as the football Robocup [78], human-robot competitions are only planed, such as the football competition projected in 2050. Recent military projects aimed at designing robotic soldiers and mobile robotic platforms equipped with weapons [79] will probably soon exhibit some ability to use their firepower against opponents according to some competitive scheme, even if ethical debate still rages over it [80],[81].

### Simulation of simple motor interactions between two humans

This section illustrates how our taxonomy can be used to implement interactive tasks using optimal control. It presents a simple simulation of two human agents rigidly fixed to a one degree of freedom pointmass that they have to move from one position to another, using various kinds of interactive behaviors described in subsection “*Taxonomy of interactive behaviors*” of the Materials and Methods. In this interactive agonistic task the two subtasks correspond to the task itself.

The interaction between two agents the dyad can be seen under a game theoretic framework. The type of interaction (game) depends on the cost of each agent and also on the coupling between them. In the case of cooperation or collaboration, when there is perfect knowledge of the state, then the problem can be transformed into an optimal control problem for each player [82], whereas in the case of antagonistic tasks, the problem can be considered as a utility-based non-cooperative game [83].

Details about the dynamics of the modelled agents, the approach used to translate the cost functions defined in the Table of Fig. 3 into a unified cost function for optimal control, as well as the couplings used in the simulations, are given in the Materials and Methods. The obtained results are presented next. Figures where obtained through the simulation of dyad dynamic interaction on MATLAB (MathWorks®) with the linear-quadratic state-feedback regulator available in the Control System Toolbox.

#### Cooperation (assistance) vs. collaboration

To implement the assistance example, we consider that the metabolic cost is much larger for the master than for the slave, and that the cost of the error of the master is high for both master and slave (see Materials and Methods for the numerical values used). On the other hand, collaboration is defined similarly to a symmetric cooperation but with a common will to reduce both errors and a similar metabolic cost for the two agents.

**Figure 6:**
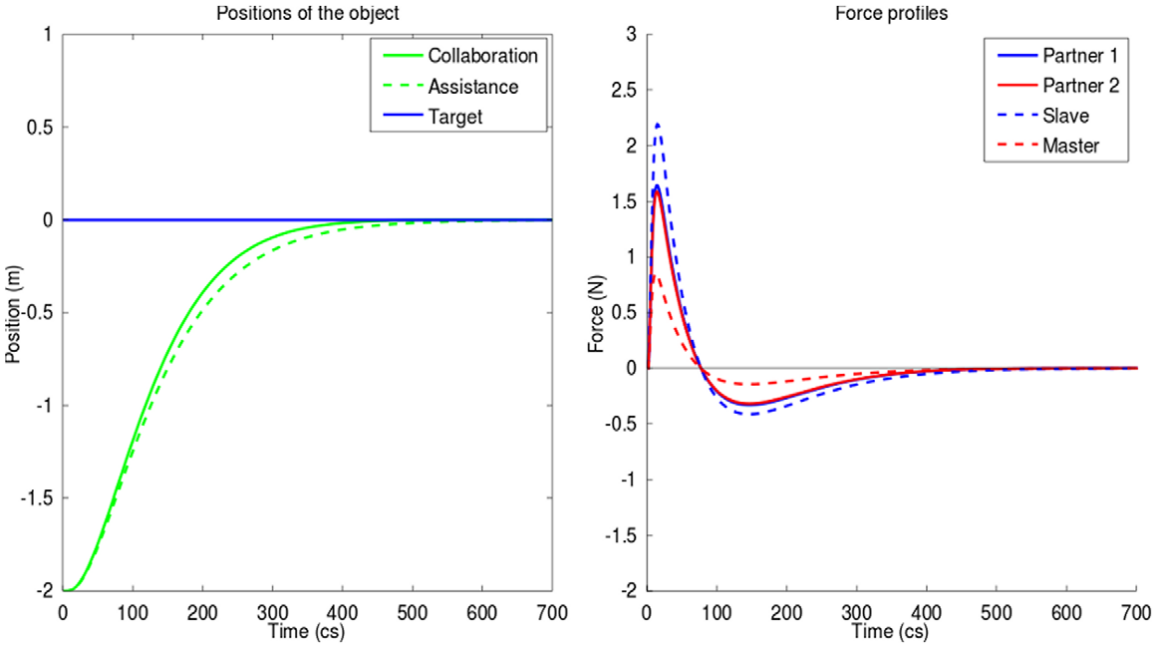
Cooperation (assistance) vs. collaboration. *Left*: the object trajectory is shown (plain line shows object displacement during a collaboration whereas dotted lines during cooperation scenario). The object is initially placed at position 2 meter away from the target that should be reached (position 0). *Right*: the forces applied by each agent on the object to make it reach the target position are shown on the right (plain lines shows force profiles applied by the each partner during the collaboration whereas dotted lines shows the force profiles applied by the master and the slave during an assistance scenario). Similar overall amount of force is needed in both cases, but the symmetric collaboration enables to reach the target faster.

Fig. 6 compares the object's movement and the force profiles for the cooperation vs. collaboration. Due to the smaller weight of metabolic cost, the slave (in dashed blue) provides most of the required amount of forces, e.g., the ratio of integrated square force is 2.7 between slave and master. Increasing the difference between both agent metabolic costs will accentuate the asymmetry in the relation, but will also tend to increase the movement duration.

In the collaboration case (solid lines in Fig. 6) the two partners' effort (i.e., the sum of the two integral of the square forces) are similar, leading to a reduction of the individual effort (i.e., integrated square force) and to a 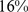 reduction of the time to reach 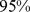 of the movement distance, relatively to the cooperation.

#### Education

The teacher attempts to concurrently minimize his effort and reduce what he perceives from student's error. Two cases were simulated: one where the student is interested in the completion of the task (higher cost of error for the student) and one where the student is lazy, thus not really interested in error minimization (low cost of error for the student) and saves his effort through a higher weight of metabolic cost (see Materials and Methods for the numerical values used).

**Figure 7:**
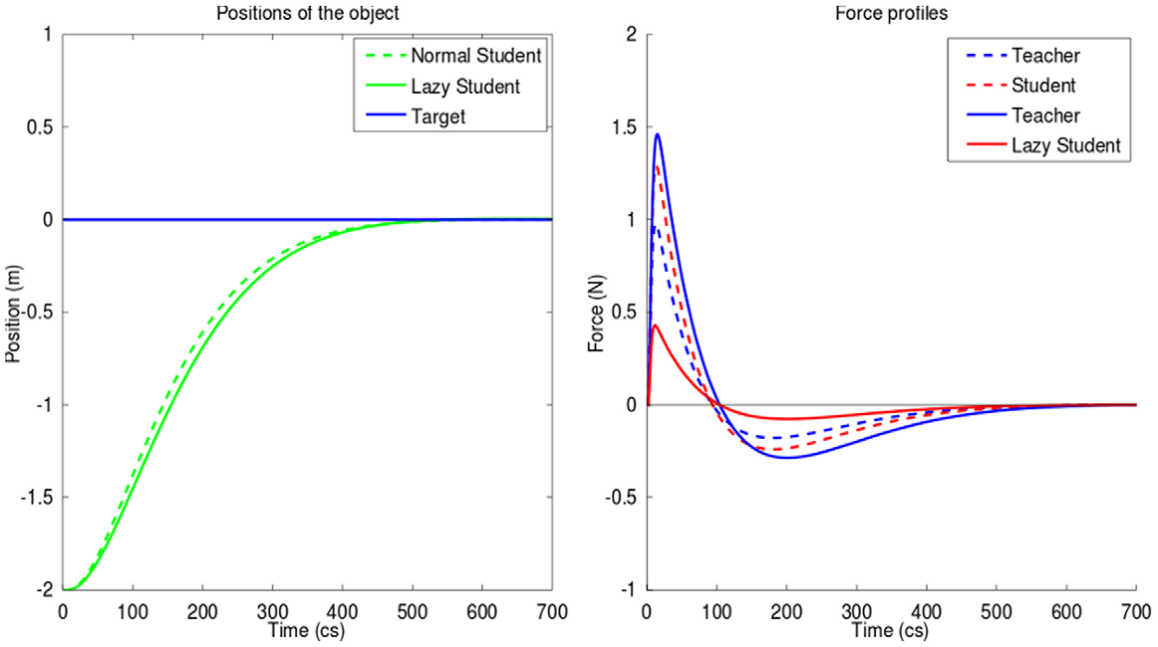
Cooperation: education scheme with hardworking or lazy student. *Left*: trajectory of the object during the two scenarios. *Right*: profiles of force applied by each subject to complete the task during the two scenarios (plain lines for the scenario where the student is lazy and mainly relies on the teacher to perform the task, dotted lines for the scenario where student is hardworking). Teacher strategy (cost function) remains the same in the two scenarios. However, although the teacher tries to minimize his involvement in the task, when he is interacting with a lazy student he is forced to provide a significant effort to bring the object on target.

Fig. 7 compares the performance obtained with the hard working student (solid lines) and with the lazy student (dashed lines). With the hard working student the teacher needs to spend only 0.75 of the student effort (measured by the integral of squared force), while with the lazy student he spends 3.59 times as much effort as the student. The movement is also 1.11 faster with the hardworking student, because the teacher refuses to behave as slave and forces the lazy student to participate.

#### Divisible antagonistic task

In this case, we simulated the *co-activity* scheme with a simple divisible task using a different target position for each agent, i.e., the subtasks are antagonistic. In order to get a clear solving of the simulation, we defined one agent to be stronger than the other through their metabolic costs (see Materials and Methods for the numerical values used).

**Figure 8:**
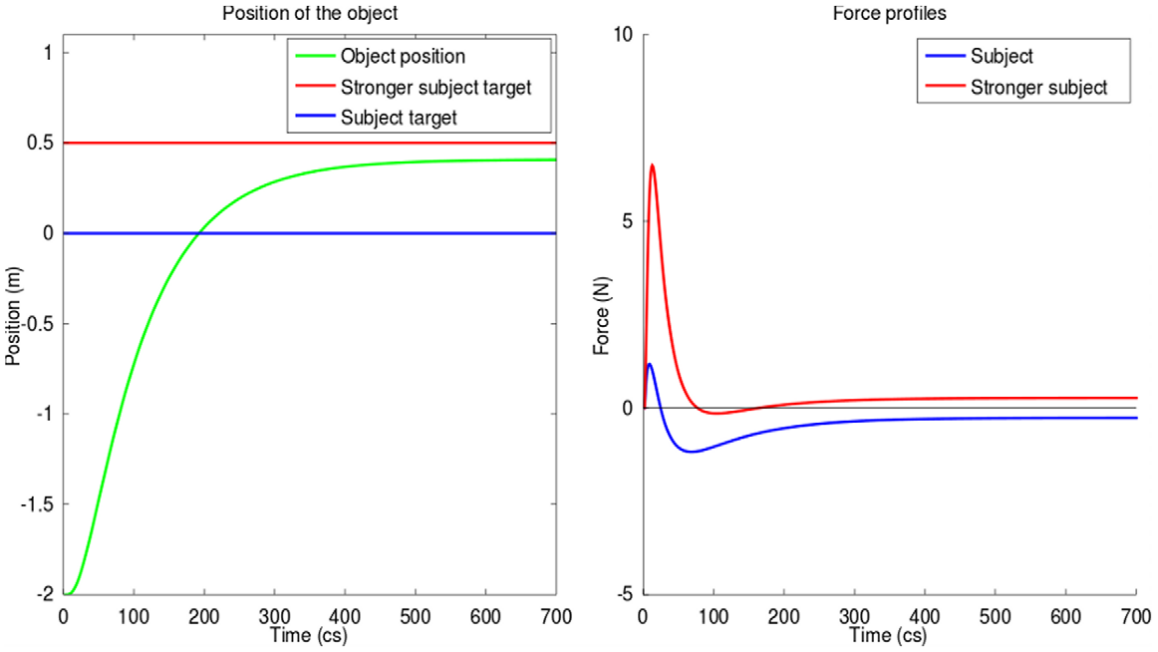
Co-activity during a divisible antagonistic task, with a subject stronger than the other. *Left*: trajectory of the object during the competition (green), with in red stronger subject's target position and in blue the weaker subject's one. *Right*: force profile applied by each subject during the completion of the task. The stronger subject (red) is able to force the other (blue) to follow him, which leads to non-zero terminal “co-contraction” (level of applied force is non-null at the end of the task).

Fig. 8 illustrates that co-activity, because of the nature of the task which is antagonistic, leads to important increase of the energetic expenditures : force levels increase (up to 7*N*) and non zero asymptotic forces appear (+/− 0.5*N*) even when one of the subject's target is reached (i.e., co-contraction), while the movement duration increases by more than 20% compared to the mean of reaching time obtained with the previous schemes.

Although the behavior may appear as a competition, this is a co-active behavior. Due to the dominance of one subject the game did not end up to an optimal solution for the system or a Nash equilibrium (a state in which none of the two agents is willing to unilaterally change her action) as could be expected in a non-cooperative game.

### Learning

This section illustrates how the cost functions determining the behavior can lead to motor adaptation, and how the taxonomy can be used to determine the control of a sport training robot step-by-step.

Considering that control is realized as the addition of feedforward (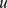) and feedback (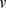) motor commands:
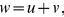
(8)we have recently derived a learning law to adapt the feedforward motor command (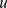) along a repeated movement [28] or in arbitrary movements [84], such as to minimize error and effort Equ.(1). For instance, if 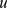 is a linear function of a parameter vector 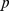, i.e.
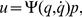
(9)(where 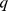 is the position vector and 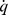 its derivative), then the gradient descent minimization of error and effort yields the learning law
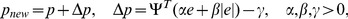
(10)which adapts the feedforward motor command as a function of error 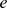. This extended nonlinear adaptive controller can be used to adapt force and mechanical impedance as demonstrated in [28], [84].

Interestingly, all the cost functions in Fig. 3 are formed of error and effort terms, so can be used to learn the own dynamic model or/and the dynamic model of the partner. For instance, if a robotic trainer is used by a human subject to learn a physical task, then the subject will likely modify his or her muscle activations according to Equ.(10) [85], [86]. If the training robot is controlled and adapted using the same laws, this will yield the education behavior in which the human will be assisted “only as needed”. Note that above cost function can be used with other learning techniques such as reinforcement learning [87].

Finally, let us now describe step by step the design of control for a sport training robot, by answering the questions of Fig. 5. The control subtasks of the user and the robot are not independent, so this is an interactive task. The sport trainer should not harm the user, so this task is agonistic. As the robot has to help the user, so their behaviors will be different and this is thus a cooperation. Finally, we have explained above that the robot should be greedy so as to yield good training, thus we are in the behavior's education type. We can thus implement above adaptive controller on the robot in order to let it promote optimal training of the human user.

## Discussion

This paper has introduced a generic framework to describe, analyze, generate and adapt motor interaction behaviors, consisting of a classification of the tasks through which subjects interact, and a taxonomy of motor interaction behaviors for two agents such as human-human, human-robot and robot-robot. In this framework, the partners' roles can be determined by answering a few simple questions as it was presented in Fig. 5. As the study of interaction of a human with the environment and between humans is complex, due to the redundancy brought by the two actors and the possible influence of conscious/high-level processes, and not much experimental material is yet available, we decided to develop this framework using an axiomatic top-down approach. However, some of the behaviors described, in particular the education behavior, are directly based on a successful computational model [25] that resulted in a novel interactive controller for robots [28]. While there are multiple ways to represent motor interaction behaviors, our taxonomy enables us to characterize a wide range of interactive strategies in a simple and extendible approach. This was illustrated by classifying existing human-robot interaction behaviors, and by generating control of typical human-human motor interactions. The concrete application of our description for the design of robot's behaviors will have to address practical issues that are out of the scope of this paper, whose goal is to define the framework and taxonomy. In particular, as for other optimization frameworks, the mathematical solution may require care of the computational aspects.

From a mathematical point of view, our framework embraces a utility-based Game Theoretic approach, using a set of cost functions to organize, understand and reproduce human motor behaviors of interactions with partners. Once the nature of the underlying task has been characterized, existence and uniqueness of a Nash equilibrium are established from Game Theory. As soon as the task has been formulated, the utility function of each player is chosen based on the assumption that the players will work towards the objective of the task, thus guaranteeing the rationality assumption of the participating players. Game Theory methods yield distributed decision making, allowing players to have different utility functions, and providing the tools to characterize the existence and uniqueness of a Nash equilibrium. It also provides tools to analyze and describe the performance of the system as a whole, though this could also be provided by alternative methods such as Lyapunov stability, contraction mapping or passivity theory. Furthermore, an analysis of the flow exchanges between subjects (using for example a Bond graph representation of the system [88]) could be used to identify the roles of each partner and thus, the nature of the interaction.

For simplicity, our framework omitted noise in the perception of own and partner's error or energy expenditure, though these may be key factors to explain switchings between multiple strategies [88] that can occur during a task completion. Similarly, we did not consider how the history of interaction may influence a current interaction behavior [89] and subject's prediction capability.

The taxonomy of this paper and its simple approach based on cost functions to describe interacting agents could also be used in different fields, other than motor interaction between a human and a robot. In neuroscience and medicine for instance, it could help interpreting pathological interactive motor behaviors (e.g., autistic behavior) through simulations of altered perception of the partners' action or one own action. Replacing “motor error” by the “market share to gain” and “energy expenditure” by “investments” could bring a clear formalism of company strategies and policies [90]. While these fields have used Game Theory, our taxonomy provides a fine characterization of the different roles which is not explicitly contained in general Game Theory. Finally, the simplicity of the adopted mathematical framework makes it suitable for use in philosophy and experimental psychology, offering computational tools for experiments, simulations and validations in the field of theory of action.

## Materials and Methods

### Simulation model

To illustrate how the cost functions of Fig. 3 can generate interactive behaviors, we simulate two human agents 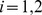 moving a pointmass 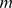 along a single axis according to the applied forces 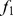 and 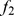. Interaction between the agents is realized through the application of forces on the rigid object.

#### Arm dynamics of one agent

A simple model of the arm dynamics can be developed by assuming that the pointmass 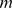 is moved along the axis by the combined action of all muscles of agent 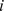 represented by the force 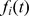, thus:
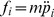
(11)

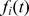
 is computed from the control signal 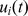 using the model of [91], where 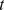 is the time. This muscle model is a second-order linear filter, that can be written as two first-order filters by using an auxiliary variable 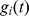


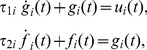
(12)where 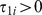, 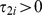 are the time constants for agent 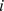. Let 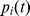 be the ‘hand’ position of agent 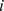 at time 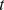, 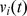 the corresponding velocity, and 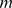 the ‘arm’ mass. Using the discrete-time transformation 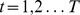


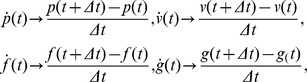
the dynamics of one agent moving the mass 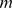 are:
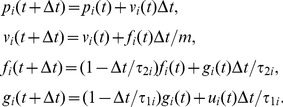
(13)Defining the error to the target 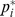 as 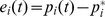, the dynamic equation 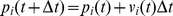 becomes:
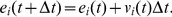
(14)Representing the current state of the discrete-time system for each agent 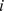 manipulating the same object as
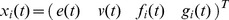
(15)(because in our simulations the different subjects are applying forces on one single rigid object and thus 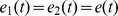 and 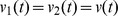), the state of each agent 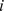 yields
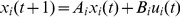
(16)with
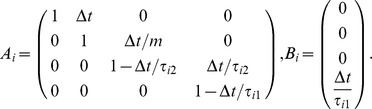
The *linear* optimal gains 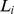 can be found via a Linear-Quadratic regulator (LQR), thus the input 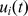 is given by
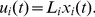
(17)For a linear system with white Gaussian noise optimal gains 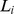 could be computed using Linear-Quadratic-Gaussian (LQG) control (though this is considered out of the scope of this paper). The cost function for each agent 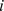 consists of the quadratic function
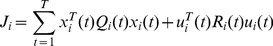
(18)where
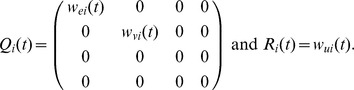
The optimal gain at time 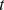 is given by
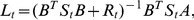
(19)where 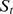 is the solution to the associated discrete-time Riccati equation:
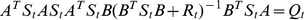
(20)provided 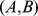 is controllable, 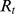 is positive definite and 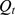 is semi-positive definite.

#### Dyad's dynamics

The state-space equation of both agent yields
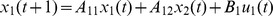


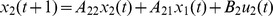
where 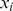 is the state vector of agent 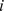 and 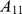, 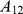, 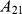, 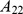 are defined below. Hence
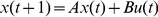
(21)with
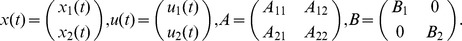
This representation allows treatment of a general class of problems with different initial positions, errors, velocities, etc. For interaction through a rigid body
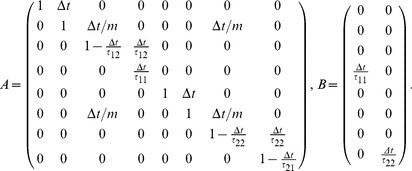
(22)In A we can identify
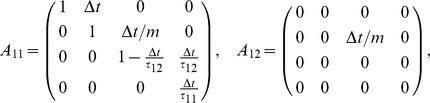


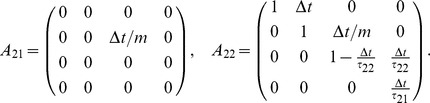
such that the interaction is realized through the non-zero component in 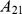 and 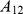. The cost function 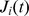 for each agent is again given by
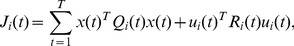
(23)where 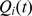 describes the kind of interaction and 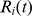 the strategy.

As aforementioned, since the task involves the interaction between two agents, a utility-based game theoretic framework could be employed in order to analyze the behavior of the agents as a dyad, and also the performance of each agent. However, in a joint cooperative or collaborative task the optimal strategy for the two agents can be determined using optimal control on the joint cost for the task's implementation.

#### Simulation parameters

The simulations shown in the Results use a mass of 1 *kg*, 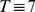 *s* and 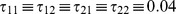. The components in the diagonal of the 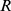 matrix were: 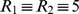. Let the cost function 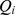 of agent 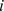 be defined as the diagonal matrix:
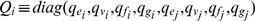
(24)with 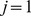 if 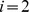 and 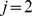 if 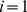.

Tuning the values of the elements of 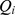 allows to directly modify the values of the gains 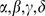 used in all the cost functions of the framework to define the different interactive kinds: 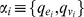, 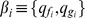, 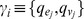 and 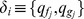. Thus, simulating the different interaction cases is performed by tuning the values of 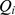.

For example, to simulate the assistance behavior, the slave motion is defined by the cost function 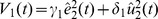 and the master behavior by 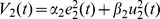. The slave, only interested in minimizing the master error and energy will thus have very small cost values for 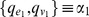 (the cost of his own trajectory and velocity error) and (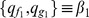 (the cost of his own force) and high cost values for 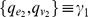 (the cost of his own trajectory and velocity error) and (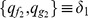. The master will only care for his own trajectory and energy and thus will have a 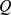 matrix characterized by null values for 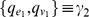 and 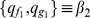.

The simulation of the divisible antagonistic task shown in the Results uses the same mathematical framework previously defined. However the simulation model is adapted to allow the use of two different errors, by adding an offset on one of the position feedback through Equ. 21.

Then, for each case:

- Assistance:
*slave:* 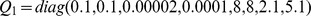

*master:* 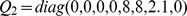

- Collaboration:
*partner 1:* 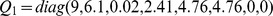

*partner 2:* 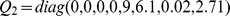

- Education:
*teacher:* 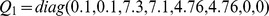

*lazy student:* 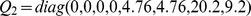

*hardworking student:* 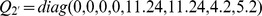

- Co-activity (divisible antagonistic task):
*subject:* 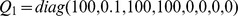

*stronger subject:* 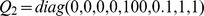

Figures shown in Results were generated by simulating the detailed dyad's dynamic on MATLAB (MathWorks®), through the use of the linear-quadratic (LQ) state-feedback regulator for discrete-time state-space system.
